# Mechanical Uniaxial Compression of 3D-Printed Non-Periodic ASA Lattice Structures Using Semi-Controlled Design Models

**DOI:** 10.3390/polym17202775

**Published:** 2025-10-16

**Authors:** Nebojša Rašović, Inga Krešić, Jasmin Kaljun

**Affiliations:** 1 Faculty of Mechanical Engineering, Computing and Electrical Engineering, University of Mostar, 88000 Mostar, Bosnia and Herzegovina; nebojsa.rasovic@fsre.sum.ba (N.R.); inga.kresic@fsre.sum.ba (I.K.); 2 Faculty of Mechanical Engineering, University of Maribor, 2000 Maribor, Slovenia

**Keywords:** semi-controlled Voronoi tessellation, stress–strain curve, mechanical response

## Abstract

This work examines the mechanical behaviour of 3D-printed stochastic lattice structures fabricated using a semi-controlled design. A primary goal is to predict and optimize the mechanical response of these Acrylic Styrene Acrylonitrile (ASA) filament structures when subjected to compressive stress. By transitioning from a purely stochastic method to a semi-controlled tessellation approach within Rhinoceros 7 software, we effectively generated the proposed design models. This methodology results in mechanical responses that are both predictable and reliable. The design parameters, including nodal formation, strut thickness, and lattice generation based on a predefined geometric routine, are associated with the regulation of the relative density. This approach aims to minimize the effect of relative density on the actual stiffness and strength evaluation. Our findings are cantered on the compressive testing of structures, which were generated using a Voronoi population distributed along a parabolic curve. We analyzed their mechanical response to the point of failure by examining stress–strain fluctuations. Three distinct behaviour stages are observed: elastic range, plastic range, and collapse without densification. The influence of crosslink geometry on the elastic responses was highlighted, with parabolic configurations affecting the peak stresses and elastic line slopes. The structures exhibited purely brittle behaviour, characterized by abrupt local cracking and oscillatory plateau formation in the plastic stage.

## 1. Introduction

The expansion of 3D printing involves the creative application of filament materials blended into additive manufacturing techniques, pushing the research investigation into material responses to external forces and developing capable structures for various applications. As one of the most rapidly advancing technologies, 3D printing depends on recent progress in materials, 3D printing hardware, geometric modelling, and design innovativeness [1]. The 3D printing industry’s growth relies heavily on progress in material science and its trends [2]. Key additive manufacturing techniques, such as fused deposition modelling (FDM) [3], fused filament fabrication (FFF) [4,5], and melting extrusion (MEX) [6,7], are advanced methods that utilize plastic filaments. Consequently, advancements in both materials and these specific technologies are vital for the field’s continued development [8]. Further development of these fusions is essential for advancing this field. Various materials are used in the 3D printing industry, and even with the significant progress made in metals, the affordability of polymer composites has opened completely fresh perspectives and possibilities for application in various fields, such as biomedicine, aerospace, and automotive [9].

Acrylic Styrene Acrylonitrile (ASA) has many applications, even much more than these three in the aforementioned fields. The ASA filament is a perfect all-purpose 3D printing thermoplastic that is suitable for various applications, as recommended by the manufacturer. It shares a similar chemical composition with ABS but outperforms it in three key areas: improved mechanical properties, superior esthetics, and exceptional UV resistance [10].

While Polylactic Acid (PLA) and Acrylonitrile Butadiene Styrene (ABS) are more commonly used in academic research due to their low cost and ease of printing [11,12,13,14], ASA was specifically chosen for this study due to its superior performance profile for engineering applications. Compared to PLA, ASA offers significantly higher thermal resistance and impact strength, making it more suitable for durable components. While chemically similar to ABS, ASA provides enhanced mechanical properties and, most critically, exceptional UV and weather resistance, which is essential for potential outdoor or long-term applications. This makes ASA an ideal candidate for creating robust, functional lattice structures intended for real-world loading conditions, justifying the focus of this investigation.

It is also notable for its high impact, chemical resistance, good moisture absorption, ability to withstand high temperatures, and excellent dimensional stability. These properties make ASA suitable for various outdoor applications exposed to the sun and other outer impacts. There is a growing trend of replacing devices composed of metals or alloys with polymers, which allows for extensive investigation of the mechanical characteristics and internal structure preparation for better customization as a replacement in a designated environment. By utilizing ASA in additively manufactured products, intricate advanced biomedical devices can be created using computer-aided design systems and design for additive manufacturing methods (DfAM) [15]. This approach is particularly beneficial for incorporating patient-specific anatomical data and designing prosthesis sockets [16].

In the last decade, there has been a significant increase in papers featuring mechanical tests of FDM samples, offering even testing conditions and optimizing methods of FDM printing parameters for improving the properties of 3D printed products [17]. Previous research has highlighted ASA’s benefits. For instance, authors in Ref. [18] investigated the mechanical properties of 3D-printed ASA, focusing on how tensile and flexural strengths are influenced by infill percentage and build direction. Their work also included a comparison between the 3D-printed parts and analogous injection-moulded components reported in the literature. In a related study, Ref. [19] sought to obtain key material data by examining specimens under five different process parameters, which demonstrated ASA’s significant tensile and flexural mechanical properties. Investigating the orthotropic properties of ASA material printed using FDM, the authors in Ref. [20] used different raster configurations of specimens, determined the elastic constants and yield properties, and showed mechanical data in various printing directions as the basis for further research interest related to this thermo polymer. The results highlighted the most suitable orientation, offering a Yield Strength of 30.5 MPa and a Young’s Modulus of 1.96 GPa as the best results. Since many papers related to polymer-based additive manufacturing of PLA and ABS explain their various applications, there has been a research space to investigate ASA’s wear strength, but also blended with other materials such as TPU, using parameters such as infill density, speed, wall thickness, raster angle, and temperature [21].

Owing to its wide application range and good mechanical properties, ASA, as an amorphous thermoplastic polymer material, is an engineering filament made to endure long periods of the sea’s open air, rain, cold water, and even saltwater. Thermoplastics are characterized by the cohesion between the polymer chains that compose them and usually exhibit orientation-dependent mechanical responses [22,23,24]. Therefore, an approach for forming or additively manufacturing a part causes it to be anisotropic or orthotropic. Thus, manufacturers always deliver printing recommendations to the user manual’s data sheets. Given the investigation of the anisotropic material behaviour of 3D printed composite structures, the authors of Ref. [25] carried out mechanical testing on specimens and revealed that build orientation significantly influenced the final properties of the material extrusion additive manufacturing method. They also provided new insights into the printing strategy–property relationship of the mechanical performance of 3D printed parts.

In light of the research and facts mentioned above, there is ongoing interest in studying the behaviour of ASA thermoplastic polymers when used in engineering applications, especially in creating lattice-structured parts, focusing on their anisotropic properties. Because the ASA integrates mechanical robustness, resistance to UV rays, and water, emphasizing an excellent surface finish, there is an investigating potential for final pieces or prototypes made with an artificial cellular philosophy application for outdoor use. The potential of cell structures is vast, but lattices stand out for their exceptional properties, such as being lightweight, strong, energy-absorbing, and vibration-reducing. Lattice structures exhibit repetition in uniform and non-uniform arrangements, which are known to be periodic and stochastic [26].

The mechanical performance of lattice structures is a subject of extensive research. Many studies have focused on periodic lattices, exploring how unit cell topology dictates properties like stiffness and strength under compression. For instance, in Ref. [27] authors investigated the crashworthiness of novel periodic lattices, highlighting the link between geometry and energy absorption. On the other side, according to Ref. [28] lattice structures were used in design and optimization of helicopter rotor blade systems to minimize vibrations. In contrast, stochastic lattices, such as the Voronoi structures in this study, are known for their isotropic properties and resemblance to natural cellular materials like bone. Recent work by Suksawang et al. [29] has further explored functionally graded stochastic lattices, showing their potential for customized mechanical responses. Our work builds upon this by introducing a semi-controlled design method that bridges purely random and fully periodic approaches, aiming to achieve predictable performance without sacrificing geometric complexity.

The mechanical properties of lattice structures can be non-uniform at the macro-design level, varying with their geometry and constituent material. Consequently, these properties are primarily dictated by three key factors: the material, the topology, and the relative density [30]. After material selection, topology is critical. It defines the nodal connectivity and the overall cell arrangement, which in turn controls the mechanical behaviour of the stochastic lattice structure [31]. Relative density, the ratio of the lattice’s physical density $\left(\rho_{ls}\right)$ to the base material’s density $\left(\rho_{m}\right)$, is also crucial and is typically managed by adjusting parameters like unit cell size, cell count, or strut thickness [32]. Many studies manipulate these factors through topological arrangement, focusing on varying the nodal connectivity [31,33]. Increased connectivity often yields more rigid structures with higher static and fatigue strengths or can be optimized for energy-absorbing applications. Advancing this, some authors [34] developed models to predict the anisotropic apparent modulus and strength using lattice density and fabric tensors, offering a valuable tool for mechanical design. In Ref. [35], a modified stochastic structure was proposed as a base for stiffness-controlled components, suitable for lightweight parts needing uniform stiffness or as biomaterials to replace trabecular bone. To determine the influence of anisotropy and infill patterns in printed polymers, researchers often use uniaxial tensile tests [30,36,37]. Compressive tests are also widely used to study the specific impact of infill patterns on the mechanical properties of lattice models.

While the mechanical behaviour of 3D-printed ASA polymers is well-documented, a research gap exists. Specifically, there is a lack of comprehensive stress–strain data for non-uniform lattice structures designed using predefined geometric routines. The behaviour of ASA under semi-controlled lattice creation conditions, where specific design patterns guide the Voronoi point distribution, remains poorly understood. A critical constraint of this research is the control of relative density. We maintain a constant Voronoi population count across all design models. This constraint is essential to isolate the effects of geometry by minimizing the significant influence that relative density typically has on the mechanical response of lattice structures. This study, therefore, aims to provide an extensive experimental and computational investigation. We will explore how the proposed geometric routine—including its geometric “fine-tuning” or smooth variations—impacts compressive strength, as reflected in significant strain plotting variations. The primary motivation is to deepen the understanding of polymer-based lattice structures, particularly those used in uniaxial compression, and to encourage further research in this area.

## 2. Materials and Methods

### 2.1. Three-Dimensional Printing of ASA Specimens

**Figure 1:**
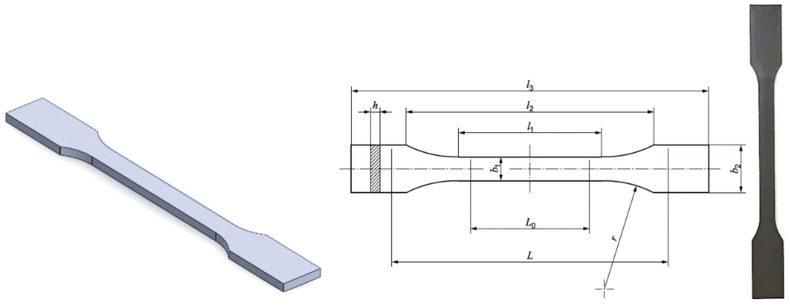
Modelled and printed testing specimen layout with geometry aligned with ISO 527-2/1A [38].

The test specimens were virtually designed in accordance with the ISO 527-2 standard [38], specifically for specimen type 1A. As detailed in Figure 1, the specimens featured an overall length $l_{3}$ of 170 mm, a width $b_{1}$ of 10 mm at their narrow portion, and a preferred thickness h of 4 mm.

For material characterization in all mechanical uniaxial tests, specimens were 3D-printed from an ASA filament using a 0.254 mm layer height. This was performed on an industrial-grade Stratasys (Eden Prairie, MN, USA) F270 (F123 series) device. The specific material was an ASA black filament (type F900-T16 Tip) manufactured by Stratasys (Eden Prairie, MN, USA). Based on its datasheet, the material properties are a yield strength ($S_{y}$) of 32.8 MPa, an elastic modulus ($E_{m}$) of 2140 MPa, an elongation at yield point ($E_{y}$) of 2.5% and an elongation at the break ($E_{b}$) of 5.9%.

All 3D printing settings were chosen based on manufacturer recommendations and standard additive manufacturing principles, using 100% infill. Due to the material’s high glass transition temperature, a heated bed was essential for optimal results. The parameters used were a printing speed of 50 mm/s, an extruder temperature of 245 °C, a bed temperature of 100 °C, and a maintained heated chamber. The extruder temperature was set 5 °C below the mid-range value to prevent initial layering issues, in line with ASA printing tips. Strict adherence to these processing values is crucial, as the manufacturer’s guidelines indicate that deviation will reduce the quality of the printed specimens. This is particularly important for avoiding high temperatures, which can cause warping, a critical issue when working with ASA. The slicer software used to prepare the printer’s route was GrabCAD Print v1.92.17.44384 (Stratasys, Eden Prairie, MN, USA). The carefully defined parameters for FDM printing are listed in Table 1.

Specimen production utilized the FDM method, which is a standardized [39] additive manufacturing process known as material extrusion (MEX). This technique dispenses a thermoplastic polymer through a hot nozzle. MEX was chosen for its significant technical capabilities, which enabled the development of the proposed design models and the subsequent test specimens. In accordance with standardized guidelines, five specimens were printed with a 100% infill structure, using only the minimal support material that the MEX technique requires.

### 2.2. Post-Printing Specimens Treatment

Because the Stratasys F270 3D printer from the F123 series was used in specimen manufacturing, in addition to the head for prime material supply (Stratasys, ASA), this method also uses an additional head for support material supply (Stratasys, QSR Support) during the building process. Thus, all specimens were dissolved in a previously prepared liquid bath for 24 h at 60 °C to remove any remaining support material.

### 2.3. Uniaxial Tests

Due to the anisotropic nature of FDM thermoplastic polymers, their tensile curves vary depending on the print orientation. We therefore tested all specimens in the printing direction, ensuring that specific stress conditions could be effectively applied to the non-uniform lattice macro design models. Before testing, every specimen was conditioned for a minimum of 40 h at 23 °C and 50% relative humidity to eliminate potential external influences and material ageing.

**Figure 2:**
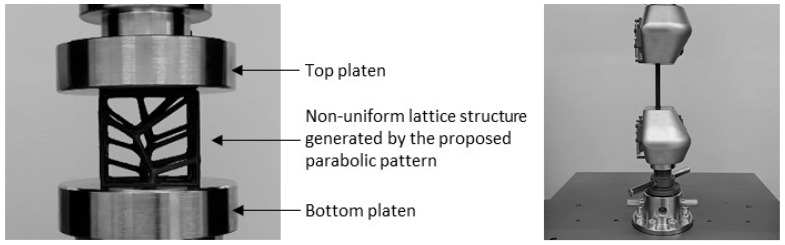
Experimental setup of the uniaxial tests using Shimadzu AGS-X.

Uniaxial tensile tests were performed on a Shimadzu AGS-X universal testing machine (Shimadzu AGS-X, Kyoto, Japan), adhering to ISO 37:2024(en) [40]. The machine was equipped with a 100 kN calibrated load cell and operated at a traverse rate of 1 mm/min. All specimen groups were analyzed under identical testing conditions. This same machine was also used for the compression tests of the non-uniform lattice structures. For the compression tests, specimens were carefully positioned on the machine platen to ensure their top and bottom surfaces were parallel and fully covered (Figure 2). This alignment guaranteed a uniform load distribution during the test. The moving platen’s speed was set to 5 mm/min, creating a quasi-static loading scenario designed to prevent any dynamic effects.

Given the tensile tests, the recorded data were obtained using the initial specimen dimensions according to ISO 527-2/1A [38], and they were recorded as engineering stress–strain data to characterize the prime material. TrapeziumX v1.5.6 material testing software (Shimadzu, Japan) was used to import standardized testing rules following ISO norms and to acquire the testing results. The uniaxial tensile testing process was set by adhering to the assigned standards [38,40], assuming that the yield ($S_{y}$) and maximum ($S_{max}$) tensile stress match (Equation (1)). In contrast, the tensile stress at break ($S_{b}$) did not match the yield ($S_{y}$) and maximum ($S_{max}$) tensile stresses (Equation (2)).

(1) $$S_{y}=S_{max}$$

(2) $$S_{y},S_{max}\neq S_{b}$$

The tensile properties were characterized and documented using symbols and abbreviations that comply with the ISO/IEC directives.

### 2.4. Finite Element Analysis Model

A Finite Element Analysis (FEA) model is a computational tool representing a specimen’s physical laws. It uses a mesh of discrete elements to approximate and solve for stress–strain variables under defined boundary conditions. The parameterization of this computational model relies heavily on the prime material’s tensile stress data, Young’s modulus, and tangent modulus of elasticity. The tangent modulus is specifically the slope of the line tangential to the flow curve at a plastic point of interest. Following the recommendations in Ref. [41], we constructed a computational model using the ANSYS v17.1 (ANSYS, Inc., Canonsburg, PA, USA) finite element-based solver. We established a multilinear isotropic hardening material model to simulate the uniaxial compression behaviour and deformation mechanisms of the lattice structure. These numerical simulations offer invaluable insights into the deformation process, aiding in the optimization of the lattice configuration for enhanced compression and energy absorption.

The finite element (FE) model consisted of a lattice structure sandwiched between a moving flat top surface and a fixed flat bottom surface. A large deflection analysis was used in the quasi-static testing to account for the significant deformation expected from the low relative density and to ensure computational efficiency. This approach acknowledges that the structure’s stiffness is not constant but varies as it moves along the load path. To handle this, the numerical parameterization involved applying the load using time stepping. The model applied the load in small increments and calculated an optimal time step at the end of each sub step based on the structure’s response to the applied forces. The simulation iterated these loading conditions until the solution converged, and the residual became acceptably small. The manufacturing process significantly influences the mechanical properties of additively manufactured lattice structures [42]. While similar “printing defects” were expected in all specimens, this study did not include these imperfections in the computational model. Consequently, the hardening model was not modified to account for any potential defects.

**Figure 3:**
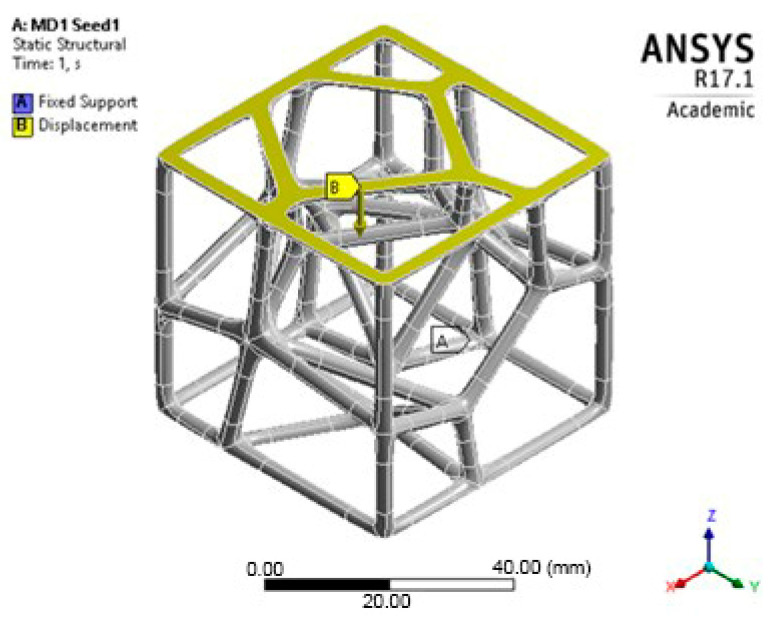
FEA boundary conditions representation.

The boundary conditions were defined to accurately simulate the experimental setup (Figure 3). The bottom flat surface was fixed with all degrees of freedom constrained. The top flat surface was constrained to move only in the vertical direction, applying a uniform compressive displacement. A displacement corresponding to the experimental loading rate of 5 mm/min was applied to the top platen. The model was meshed using tetrahedral elements (SOLID187). A bonded contact was defined between the lattice and the platens to prevent sliding. A mesh sensitivity analysis was performed by refining the mesh density until the change in peak reaction force between iterations was less than 2%, ensuring that the numerical results were independent of the mesh size.

A multilinear isotropic hardening model was developed to distinguish the material’s behaviour into two independent effects: elastic and plastic. This method enables the differentiation of temperature-dependent datasets. In this model, the elastic modulus significantly influences the initial region, while the plastic strain-stress behaviour describes the response after the yielding process begins.

### 2.5. The Proposed Design Model for Lattice Structure Generation

A deeper understanding of the mechanical behaviour within lattice structures is necessary, especially considering the principles of deflection, the bending-dominated nature of the components, and the impact of relative density. Typically, Voronoi tessellation is used to generate a randomized arrangement of cells within a set volume, creating an open-cell lattice known as a stochastic structure. However, this method offers the user no control over the cell arrangement beyond defining the initial generation parameters.

**Figure 4:**
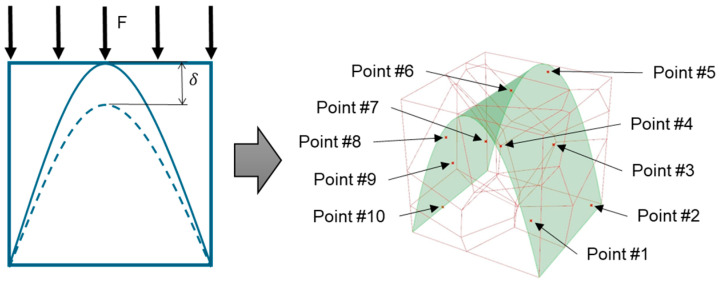
The Voronoi tessellation’s randomized point distribution was geometrically founded on a parabolic pattern.

This study’s hypothesis was that implementing a predetermined geometric routine to arrange the cells would provide a semi-controlled mechanism for point distribution. We created stochastic lattices in Rhinoceros 7 (Robert McNeel & Associates, TLM, Inc., Seattle, WA, USA) by populating a 50 × 50 × 50 mm^3^ cuboidal volume with such semi-controlled, pseudo-randomly distributed points. Unlike purely stochastic methods, these structured arrangements are intended to anticipate deformation and produce a more consistent, dependable mechanical response. Our proposed approach used a parabolic pattern as the geometric foundation for this randomized point distribution in the Voronoi tessellation (Figure 4).

In line with the intended compressive loading scenario for these lattices, we proposed a method to control the relative density. This was achieved by keeping the Voronoi population count, and thus the number of struts and nodes, constant across the different orientations (Seeds) of the parabolic pattern (Figure 4). This control is vital because it is crucial to consider mechanical strength criteria in correlation with relative density. The Gibson-Ashby model [43] is the foundational starting point for most papers addressing this issue. In this model, a remote compressive stress creates a force ($F≅\sigma L^{2}$) on the cell edges, causing them to bend and leading to a specific bending deflection (*δ*):

(3) $$\delta =\frac{FL^{3}}{E_{m}I}$$

Equation (3) relates to a horizontal strut of length L that is loaded at its midpoint by a force F, causing it to deflect. In this expression, $E_{m}$ is the modulus of the solid material from which the strut is made, and I represents the second moment of area for the cell edge’s circular cross-section. The approximate compressive strain (ε) experienced by the entire cell is $\varepsilon =\frac{2\delta }{L}$. Combining these values yields the following relationship:

(4) $$\frac{E_{ls}}{E_{m}}={\left(\frac{\rho_{ls}}{\rho_{m}}\right)}^{2}$$

Equation (4) comprises the equality of the relations between two elasticity moduli (elasticity modulus of lattice structure, $E_{ls}$ and solid material, $E_{m}$) and two relative densities squared up (relative density of lattice structure, $\rho_{ls}$ and solid material, $\rho_{m}$).

In a bending-dominated structure (such as open-cell lattices), the struts or cell walls are not exposed to loads through direct stretching/compression contact. Instead, they bend under applied remote loads. The stiffness of a bent beam is governed by its second moment of area (with strut thickness *t*). Since the relative density scales as ${\left(\frac{t}{L}\right)}^{2}$, (according to the Ashby–Gibson model, which predicts that cellular solids are characterized by their relative density, defined as the ratio of strut thickness and length), the effective modulus ends up scaling as ${\left(\frac{t}{L}\right)}^{4}$, which is proportional to ${\left(\frac{\rho_{ls}}{\rho_{m}}\right)}^{2}$. Thus, Equation (4) tells us that in a bending-dominated lattice, stiffness decreases with the square of relative density. That is the central assumption from which we began creating equally tessellated volumes (with the same number of “count” command input and holds on relative density at an unchanged value for all lattice designs), but varying position of the parabolic surface for seed examination and final product design implementation.

**Figure 5:**
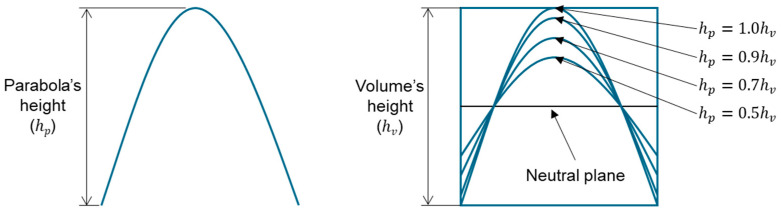
The routine for distributing points within the tessellation was determined by the major geometric dimensions, specifically the volume’s height ($h_{v}$) and the parabola’s height ($h_{p}$).

The hight of the parabola ($h_{p}$) is a geometric entity extruded and positioned inside predefined volume dimensions, which could be any volume height, width, or depth, but for the paper’s aim, determined in four primary designs varying only its height ($h_{p}$) concerning the volume height ($h_{v}$), and are following: 0.5$h_{v}$, 0.7$h_{v}$, 0.9$h_{v}$, and 1.0$h_{v}$ (Figure 5).

Following the proposed semi-controlled design model, lattice structures were modelled in a 125 cm^3^ cuboidal framework, generating each design using 10 points in randomly distributed positions over the parabolic pattern, thus making one seed orientation. The population arrangement in one-tessellation changes for each seed switch (point orientation). Two seed orientations (S1) and (S2) were used in the 10-point random distribution along a parabolic pattern (Table 2).

Considering the data in Table 2, the semi-controlled mechanism bypassed the uncontrolled tessellation of the lattice constituents, thereby significantly affecting the uniform values of the lattice density for each primary type of parabolic pattern.

## 3. Results and Discussion

### 3.1. Uniaxial Tensile Tests of Specimens

This subsection identifies the prime material characterization from which the lattice designs were made as a crucial step in establishing a computational model for evaluating its mechanical uniaxial compression behaviour. Uniaxial tensile tests were performed on solid ASA specimens to determine the base material’s intrinsic mechanical properties. This data, particularly the full stress–strain curve, was essential for developing and parameterizing the accurate multilinear isotropic hardening material model used in the subsequent Finite Element Analysis (FEA) of the lattice structures.

**Figure 6:**
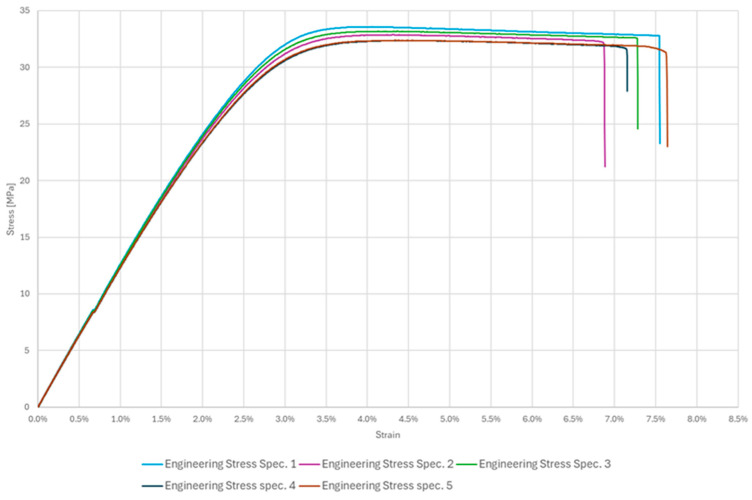
Stress–strain curves for ASA specimens.

As expected, Figure 6 shows a typical engineering stress–strain curve distribution for thermoplastic polymers that is dependent on the 3D printing settings used in specimen printing. Three characteristic zones of ASA were shown: the elastic range up to the point of over-yielding, the slight nonlinear increase in stress with an unclear distinction between yielding and maximum tensile stress, and the subsequent deformational softening of the material. The experimental tensile test delivered an average yield stress of 32.87 MPa, because of the nature of the thermoplastic polymer, it matched the maximum tensile stress. In addition, it matches the manufacturer’s previously stated material datasheet. All significant tensile stress points lie in the range of 3.2–3.7% of strain, and all had uniform elongation behaviour in the yielding area. Elongation at the breakpoint ($E_{b}$) yielded inconsistent results for several reasons. The lack of automatic control over room temperature and humidity affected the results, as the elongation at break tended to increase with temperature.

Additionally, the tested thermoplastic polymer did not exhibit significant ductile characteristics, and imperfections within the specimen’s internal structure stemming from additive manufacturing also contributed to the variation in the results. Although the elongation at break results may seem varied and inconsistent, it is essential to note that this variation is to be anticipated when considering previously described reasons. Considering the initial settings’ parameters of printing, which bound the testing and varied in each paper, the results align with findings from earlier studies on characterizing 3D-printed ASA [18,19,20].

The yield strength (*S_y_*) was determined as the maximum stress (*S_max_*) on the engineering stress–strain curve, as the material did not exhibit a distinct yield point, which is a common practice for such thermoplastics.

### 3.2. Uniaxial Compression Tests of Proposed Lattice Structures Designs

**Figure 7:**
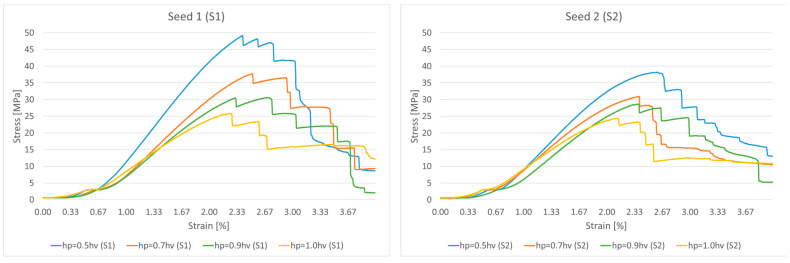
Mean approximated stress–strain curves for different parabolic designs.

Uniaxial compression tests were conducted on the 3D-printed ASA non-uniform parabolic-based lattice structure specimens. Following the approach described in Section 2.5, we modelled the lattice structures for experimental testing within a 125 cm^3^ cuboidal volume, measuring 50 × 50 × 50 mm. For testing purposes, each of the four primary designs was printed in a series of five lattice structures, varying in two orientations (S1 and S2). All parabolic lattice specimens were compressed to the failure of the ultimate structures and reached the total collapse stage. The graphs in Figure 7, which display the mean curve approximation for each design, confirm that all structures follow a typical stress–strain response. This response shows three distinct stages: an elastic range where yielding and maximum tensile stress are not clearly distinguishable; a plastic range, characterized by a nonlinear decreasing stress plateau; a final collapse stage that occurs without densification. Strut walls underwent small deflections at the onset of the compression process, displaying a linear elastic response. During this initial phase, the strut rows deformed uniformly, and the stress value increased steeply with deformation until it reached a peak stress.

**Figure 8:**
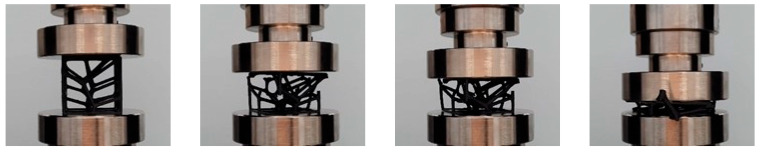
Different deformation stages of the compressed structures.

As Figure 7 clearly shows, structures with different parabolic tessellation parameters produced varied slopes in their linear responses and different peak stress values. This highlights how significantly the crosslink geometry influences the structures’ elastic response. The lattices created with a parabolic surface distribution spanning one-half the volume’s height ($h_{p}=0.5h_{v}$) exhibited particularly pronounced peak stresses and elastic line slopes. Once the peak stress is reached, localized deformation begins in the form of plastic yielding, which creates a “zigzag” deformation layout and involves significant brittle fracture within specific rows of the structure. This local deformation is caused by the bending of diagonal struts, which forms plastic “elbows” or yielding at the connecting nodes. The initial collapse causes the stress value to drop, marking the beginning of a plateau formation characterized by a nonlinear stress decrease. After a row of struts is fully deformed, the adjacent struts make contact, causing that row to stiffen; this is recorded in the charts as the straight-line portions of the zigzag plateau. The deformation then propagates to adjacent layers, leading to a progressive collapse of the structure. As shown in Figure 8, the responses during the plateau stage differ for each structure, further confirming that the varying parabolic configurations affect the mechanical behaviour. The process concludes when all strut rows collapse, with the struts colliding into a pile; the charts show a complete absence of a densification stage. Based on their compressive behaviour and stress–strain response, the results confirm that the proposed ASA designs can be classified as brittle structures. They present an oscillatory plateau caused by brittle crushing, consistent with the findings in Refs. [44,45] for Voronoi tessellations. Furthermore, in line with expert analysis of polymer stress–strain curves [46], the data shows noisy fluctuations during the plastic flow stage, which arise from abrupt local cracks.

Because there is no steep increase in the stress response in the third chart stage, indicating the absence of any densification, it results in a limited energy absorption capability. Because no densification point was recorded (no upward stress–strain flow trajectory in the third stage), it was confirmed that adjacent strut rows collapsed progressively, considering the relative density.

Upon thoroughly analyzing the stress–strain data recorded and presented in the two charts, it is possible to confidently distinguish between the four primary designs based on the hardness of the structure, determined by their initial slopes. The parabolic design dimension of one-half-volume height ($h_{p}=0.5h_{v}$) has the steepest (highest) slope, accounting for both seed distributions, which indicates the greatness of hardness. The fact that the hardness of the structures decreases linearly as the proposed dimensions of the primary designs also change, where following the analogy, it comes to the fact that a lower hardness characterizes the full height of the volume parabolic design because the slope is the lowest. Nevertheless, this does not imply that the full-volume height parabolic design ($h_{p}=1.0h_{v}$) significantly derives weaker structures because, on the contrary, the charts show a more extended break elongation plateau for them, confirming that these designs can emerge with a better stress-hardening response, resulting in toughness features in the structures. Given these recorded facts, and referring to Ref. [47], parabolic designs with a one-half-volume height ($h_{p}=0.5h_{v}$) are brittle and hard, whereas the full-volume height parabolic designs ($h_{p}=1.0h_{v}$) are soft and slightly tenacious. A thorough review of the mechanical characteristics of the proposed design is provided in Table 3.

Figure 8 illustrates the various deformation stages of the compressed structures as a function of deformation percentage. During compression, some crosslinks within the structure experienced buckling deformation, which was an expected outcome considering the relative density and the orientation of certain struts. Additionally, some structural components underwent shifting deformation, causing struts to move sideways. This shifting behaviour is common for non-uniform lattices at high strains and generally leads to a loss of the global arrangement, resulting in a specific nonlinear mechanical response.

The most important observations that can be derived from the stress–strain recorded data are stress peaks with no clearly indicated distinction between yield points concerning the maximal tensile stress, regardless of which of the four primary designs the curves represent. The peak stress exhibited a broad variance in both seed distributions, with values reaching 48 MPa for the parabolic tessellation design of one-half-volume height to 25 MPa for the full-volume height design in the first seed (S1). In the case of the second orientation (S2), the peak stress varies in a smaller range than the first, reaching 38 MPa at a maximum to 24 MPa at a minimum. Most importantly, both orientations (S1 and S2) resulted in equivalent lattice structure responses, ranging designs according to the data of the charts in the same order, confirming the reliability of the proposed models assembled by the semi-controlled tessellation mechanism.

In summary, the mechanical response of stochastic structures to varying geometric parameters of the parabolic distribution within tessellated models is highly dependent on them. The proposed semi-controlled mechanism of the stochastic population showed a reliable and repeatable approach, guaranteeing the predictable behaviour of the lattice macro-design when it comes to product application for weight-light design purposes. In addition, the proposed approach ensures an improved mechanical response to the outer loading conditions by selecting the appropriate parabolic design parameters.

### 3.3. Uniaxial Compression Testing over Parameterized Computational Model

The parameterized computational model was used to validate experimental data from compression tests and to confirm the nature of the responses of the structures, as described previously. The obtained stress–strain data as the result of the prime material tensile characterization were used to establish the multilinear isotropic hardening model as a foundation of the computational model to determine the compressive response in differently generated non-uniform lattice macrostructures based on the proposed semi-controlled designs presented in Section 2.5. In the simulation, reaction forces were measured in the upper region of the lattice structure. These forces were then numerically correlated with the displacement of the upper contact surface as it moved relative to the fixed, immovable bottom surface.

**Figure 9:**
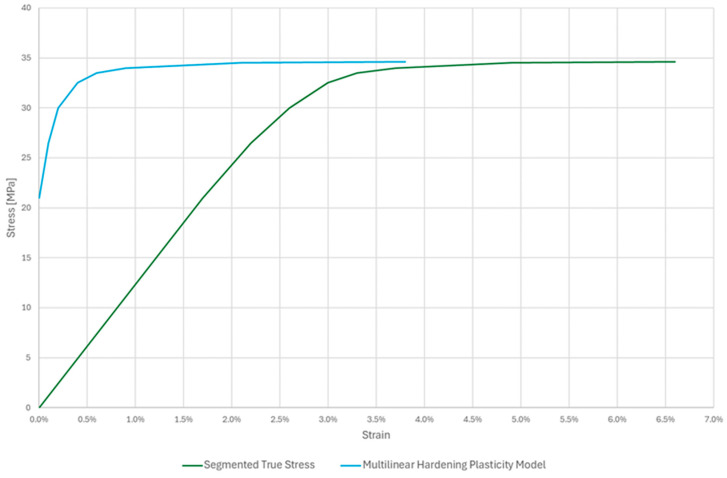
Mean Strain-Stress curves and multilinear plasticity hardening model.

Finite Element Analysis (FEA) was used to simulate the lattice deformation behaviour and extract quasi-static compressive responses. The multilinear isotropic hardening model was used to construct a nonlinear explicit simulation to evaluate the mechanical uniaxial compression of 3D-printed non-periodic ASA lattice structures generated via four primary parabolic distribution patterns inside the predefined cuboidal volume. The model represents the stress–strain relationship as a series of linear segments. Each segment is defined by a tangent modulus, a method that accurately captures the lattice’s strain hardening. To create this multilinear plasticity hardening model, the true mean stress–strain curve was segmented, as illustrated in Figure 9.

The established parameterized FE model employs a sophisticated loading method that operates across multiple time steps. Instead of applying the full load at once, the model gradually adjusts the load incrementally as the solution progresses. This time-stepping approach allows the simulation to carefully track the structure’s response at each stage of the deformation process. The primary advantage of this incremental method is that it ensures a smooth force-displacement curve is obtained. By applying the load in a series of smaller, controlled steps, the model can accurately capture the nonlinear behaviour of the material without encountering numerical instability. This gradual application more closely mimics a real-world quasi-static test and is essential for modelling the changing stiffness of the structure.

**Figure 10:**
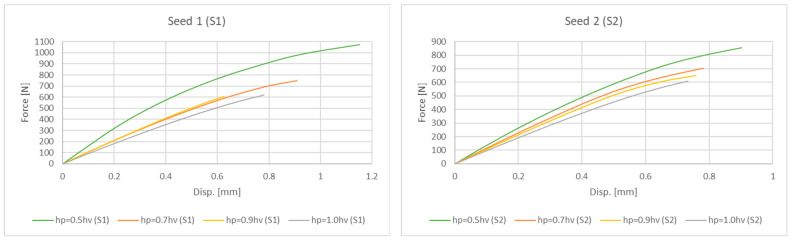
Force-Displacement curves derived by FE analysis for both orientations S1 and S2.

Ultimately, this methodical approach results in more precise and reliable numerical charts and data. The smooth curve generated through incremental loading provides a high-fidelity representation of the structure’s mechanical response. This increased precision is clearly demonstrated in the results presented in Figure 10.

Considering the data plotted in Figure 10, it is clear that the parabolic design with a one-half-volume height ($h_{p}=0.5h_{v}$) ensured the most vigorous response, achieving the highest force values and confirming the named hardness feature as the most relevant and correlative for this structure design. Similarly to the experimental results, the full-volume height parabolic designs ($h_{p}=1.0h_{v}$) again showed the lowest slope and measured the weakness force values, expressing the softness in their mechanical response and confirming the nature of the structures already described. These results demonstrate that the position and size of the geometric parabolic surface, which serves as the basis for the Voronoi population distribution, directly affects the mechanical response of the structures. Furthermore, it ensures high reliability and repeatability for achieving predictable mechanical behaviour in modelling the macro design in global product assembly.

**Figure 11:**
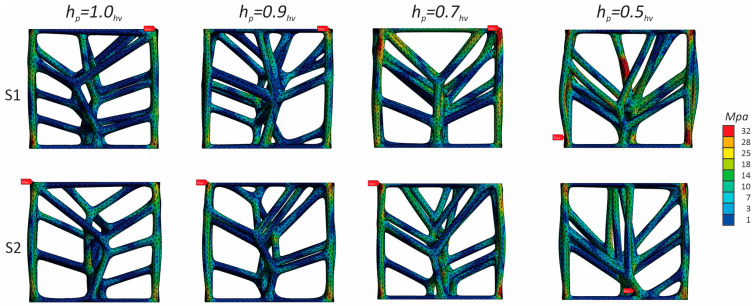
Stress–displacement plots at 2% strain derived by FE analysis for both orientations S1 and S2.

To better understand the deformation mechanisms, the von Mises stress distributions at 2% strain are presented in Figure 11. The analysis reveals that for all designs, stress concentrations appear at the nodal junctions and in the slender, vertically oriented struts. In the *h_p_* = 0.5*h_v_* design, these stresses are distributed across a more robust lower structure, contributing to its higher stiffness. This confirms that failure initiates via brittle fracture at these high-stress points, aligning with the experimental observations.

To quantitatively evaluate the FEA model, the compressive modulus predicted by the simulation was compared against the mean experimental values, as shown in Table 4. The numerical results show strong agreement with the experimental data, with a maximum deviation of less than 8%, thus validating the predictive capability of the FEA model.

### 3.4. Significance of the Plateau Region in Compression Testing of Stochastic Structures

The plateau region of the stress–strain curve is a critical indicator of energy absorption and structural resilience when characterizing lattice structures under uniaxial compression. This phase, which typically occurs after the peak stress and before densification (if present), reflects the progressive collapse of the individual cells within the lattice. For stochastic structures, where irregularity governs cell arrangement and nodal connectivity, the plateau is often oscillatory owing to localized failure mechanisms and heterogeneous deformation behaviour.

In the present study, the plateau region was notably pronounced in all tested designs, characterized by a series of abrupt stress drops followed by partial recoveries, corresponding to brittle fracturing and localized plasticity at nodal intersections. These fluctuations are intrinsic to the stochastic nature of tessellation, where the struts fail sequentially rather than uniformly. The presence and morphology of the plateau provide crucial insights into the failure mode, indicating whether the deformation is stable, energy-dissipating, abrupt, and catastrophic.

The mechanical relevance of the plateau lies in its potential applications involving impact mitigation, vibration damping, and structural buffering. Specifically, in safety-critical applications, such as helmet padding, protective casing, or prosthetic liners, the ability of a material to sustain deformation over an extended strain range while maintaining moderate stress levels is highly desirable. In such contexts, the plateau region represents a controllable energy sink, and its optimization via design parameters such as strut orientation, relative density, and tessellation geometry can significantly enhance the functional performance of the structure.

The findings confirm that by introducing semi-controlled geometrical routines in stochastic lattice design, not only can the peak mechanical properties be influenced, but the behaviour throughout the plateau phase can be tailored for specific energy-absorbing purposes. Therefore, the plateau region must be treated not as a transitional phase, but as a design-relevant mechanical signature of stochastic lattice structures.

### 3.5. The Elastic Plateau in Stochastic Lattice Compression

**Figure 12:**
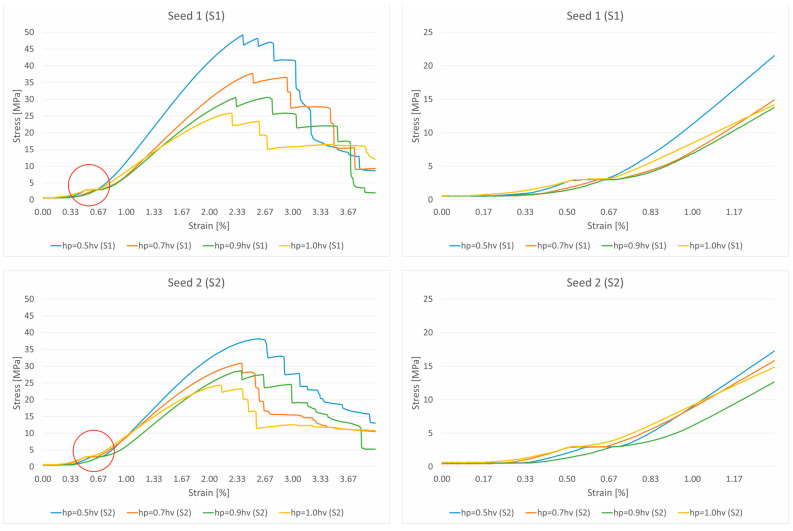
Elastic plateau on approximated stress–strain curves for different parabolic designs.

The initial phase of the stress–strain response recorded in the experimental curves for all the tested lattice configurations revealed a short but distinguishable elastic plateau prior to the attainment of the peak stress (Figure 12). Unlike the idealized linear elastic behaviour of homogenous solids, this quasi-horizontal segment suggests that even within the elastic regime, the stochastic lattice structure accommodates small deformations through localized micro-adjustments before the global stiffness is mobilized.

This behaviour can be attributed to several structural and material factors. First, the inherent irregularity of semi-controlled Voronoi tessellation leads to an uneven stress distribution across the struts, delaying uniform elastic engagement. Some struts undergo bending or slight rotational displacements at nodal connections under low loading, which does not immediately translate into a proportional increase in global stress. Second, micro-gaps or imperfections at junctions resulting from FDM printing may introduce short-range compliance until the contact is fully established across all structural paths.

From a mechanical standpoint, this initial elastic plateau indicates a brief compliance phase, in which the structure adapts without notable resistance before transitioning to a stiffer, fully load-bearing state. It serves as an important indicator of the lattice accommodation capacity under minimal stress and may be exploited in applications where low-stress isolation or shock initiation buffering is desirable. In scenarios, such as wearable orthoses or packaging systems, this elastic delay can reduce the transmission of sudden forces to the underlying structures.

The repeatability of this behaviour across all tested configurations, particularly those with lower *h_p_* values, reinforces its association with the tessellation geometry and printing-induced microstructural features. While its duration and magnitude are relatively minor compared to those of the main plastic plateau, its presence affirms the complex multiphase response of stochastic lattices and merits further parametric exploration in future research.

## 4. Conclusions

This research provides a detailed investigation of the mechanical behaviour of non-periodic ASA lattice structures generated through a semi-controlled Voronoi tessellation approach. By employing a predefined parabolic routine for the point distribution, this study successfully moderated the uncontrolled variability of stochastic lattices, enabling the development of structurally predictable and mechanically consistent lattice configurations. Experimental uniaxial compression tests demonstrated that the geometric parameter *h_p_*, representing the height of the parabolic surface used in tessellation, significantly influenced structural performance, particularly in terms of stiffness, peak stress, and post-yield behaviour.

All the structures exhibited a brittle mechanical response with distinct elastic, plastic, and collapse phases, and the absence of densification indicated a limited energy absorption capacity. The most rigid and peak stress-resilient configuration was observed in lattices derived from the *h_p_* = 0.5*h_v_*; design, whereas the *h_p_* = 1.0*h_v_* configuration offered extended deformation prior to failure, indicating a modest enhancement in toughness.

To mitigate the observed brittle behaviour, future work could explore several strategies. Material modifications, such as creating blends of ASA with a more ductile thermoplastic like TPU, could enhance toughness. Geometrically, incorporating fillets at nodal junctions would reduce stress concentrations, while designing topologies that favour a bending-dominated deformation mechanism over the current stretching-dominated one could promote more graceful failure.

Finite-element simulations using a multilinear isotropic hardening model effectively replicated the experimental trends, further validating the reliability of the proposed design methodology.

Overall, the presented semi-controlled tessellation strategy is a viable method for generating mechanically optimized application-oriented stochastic lattice structures. This approach holds substantial promise for future research and development of lightweight, energy-efficient components across automotive, biomedical, and outdoor structural applications, particularly where predictable performance under compressive loading is essential.

The findings have direct implications for specific end-use applications. For instance, in the design of custom prosthetic sockets, our semi-controlled method could be used to spatially grade the lattice stiffness. Areas requiring high structural support could use the rigid *h_p_* = 0.5*h_v_* configuration, while areas interfacing with sensitive tissue could employ the more compliant *h_p_* = 1.0*h_v_* design for enhanced comfort, all within a single, lightweight component.

While this study focused on uniaxial compression, we acknowledge that this represents only one loading condition. Future investigations should include multi-axial loading and fatigue tests to fully characterize the durability and performance of these structures under more complex, real-world service conditions. Such data would be invaluable for validating these structures for safety-critical applications.

## Figures and Tables

**Table 1:** Printing parameters of testing specimens.

Parameter	Value
Layer height	0.254 mm
Infill	100%
Printing speed	50 mm/s
Extruder temperature	245 °C
Bed temperature	100 °C
Heated chamber	Enabled

**Table 2:** Relative density by 10-point random distribution along the parabolic pattern.

Type of Parabolic Pattern (h)	S1 Relative Density ${(\rho }_{ls})$	S2 Relative Density $\left(\rho_{ls}\right)$
$h_{p}=1.0h_{v}$	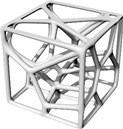 6.752%	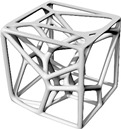 6.756%
$h_{p}=0.9h_{v}$	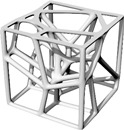 6.868%	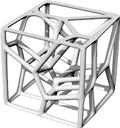 6.824%
$h_{p}=0.7h_{v}$	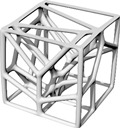 6.939%	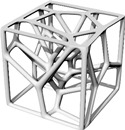 6.914%
$h_{p}=0.5h_{v}$	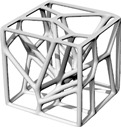 7.020%	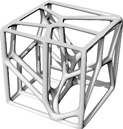 7.302%

**Table 3:** A thorough review of the mechanical characteristics of the proposed designs.

Mark	Parabolic Designs	Peak Stress [MPa]	Compressive Modulus (MPa)	Strain at Peek Stress [%]
a.	$h_{p}=1.0h_{v}$	26.26 ± 3% (S1) 24.05 ± 6% (S2)	16,090 ± 2% (S1) 16,390 ± 6% (S2)	2.40 ± 5% (S1) 2.27 ± 6% (S2)
b.	$h_{p}=0.9h_{v}$	30.43 ± 6% (S1) 28.56 ± 7% (S2)	19,600 ± 7% (S1) 18,750 ± 4% (S2)	3.05 ± 2% (S1) 2.47 ± 5% (S2)
c.	$h_{p}=0.7h_{v}$	36.48 ± 7% (S1) 30.45 ± 1% (S2)	21,840 ± 6% (S1) 19,580 ± 1% (S2)	2.32 ± 6% (S1) 2.37 ± 1% (S2)
d.	$h_{p}=0.5h_{v}$	48.24 ± 6% (S1) 35.75 ± 7% (S2)	28,450 ± 9% (S1) 24,450 ± 5% (S2)	2.36 ± 7% (S1) 2.40 ± 7% (S2)
		a<b<c<d 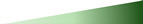	a<b<c<d 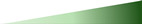	a>b>c>d 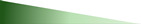

Note: Compressive Modulus has been calculated as the slope of the Strain-Stress curve in range form 1% to 1.8%.

**Table 4:** A comparation of experimental and numerical compressive modulus.

Mark	Parabolic Designs	Experimental Compressive Modulus (MPa)	Nuzmerical Compressive Modulus (MPa)	Deviation (%)
a.	$h_{p}=1.0h_{v}$	16,090 (S1) 16,390 (S2)	16,050 (S1) 16,750 (S2)	1% (S1) 2% (S2)
b.	$h_{p}=0.9h_{v}$	19,600 (S1) 18,750 (S2)	19,100 (S1) 18,200 (S2)	3% (S1) 3% (S2)
c.	$h_{p}=0.7h_{v}$	21,840 (S1) 19,580 (S2)	21,250 (S1) 19,150 (S2)	3% (S1) 2% (S2)
d.	$h_{p}=0.5h_{v}$	28,450 (S1) 24,450 (S2)	26,300 (S1) 23,250 (S2)	8% (S1) 5% (S2)

## Data Availability

The data supporting the findings of this study are published in a data repository (https://doi.org/10.5281/zenodo.17011614), and available from the corresponding author upon reasonable request.
